# How do interventions to improve the efficiency of acute stroke care affect prehospital times? A systematic review and narrative synthesis

**DOI:** 10.1186/s12873-022-00713-6

**Published:** 2022-09-03

**Authors:** Graham McClelland, Sarah Hepburn, Tracy Finch, Christopher I. Price

**Affiliations:** 1 Stroke Research Group, Population Health Sciences Institute, Newcastle University, Newcastle upon Tyne, NE2 4HH England; 2 North East Ambulance Service NHS Foundation Trust, Bernicia House, Goldcrest Way, Newburn Riverside, Newcastle upon Tyne, NE15 8NY England; 3 Department of Nursing, Midwifery & Health, Northumbria University, Room B126 Coach Lane Campus West, Newcastle upon Tyne, NE7 7XA England

**Keywords:** Prehospital, Stroke, Times

## Abstract

**Background:**

Emergency medical services (EMS) are the first point of contact for most acute stroke patients. EMS call to hospital times have increased in recent years for stroke patients in the UK which is undesirable due to the relationship between time and effectiveness of reperfusion treatment. This review aimed to identify and describe interventions devised to improve the efficiency of acute stroke care which reported an impact on ground-based EMS call to hospital times.

**Methods:**

A systematic review of published literature identified from five databases (Medline, EMBASE, CINAHL, the Cochrane library and the Database of Research in Stroke (DORIS)) from January 2000 to December 2020 with narrative synthesis was conducted. Inclusion criteria were primary studies of ground-based EMS, focused on stroke and aiming to improve EMS times. Papers published before 2000, focussing on mobile stroke units or in languages other than English were excluded. Two reviewers independently screened prospective titles. Cochrane ROB2 and ROBINS-I tools were used to assess for risk of bias. This review was funded by a Stroke Association fellowship.

**Results:**

From 3767 initial records, 11 studies were included in the review. Included studies were categorised into three groups: studies targeting EMS dispatch and EMS clinicians (*n* = 4); studies targeting EMS clinicians only (*n* = 4); and studies targeting whole system change (*n* = 3). Suspected stroke patients were the primary population studied and most (*n* = 10) interventions involved clinician education. Only one study (9%) reported a significant decrease in call to hospital time in one subgroup whereas two studies (18%) reported a significant increase in call to hospital time and all other studies (73%) reported no significant change.

**Conclusions:**

Based on the included studies, interventions intended to improve the efficiency of the acute stroke pathway rarely improved EMS call to hospital times. Included studies were heterogenous and rarely focussed on the review topic which limits the usability of the findings. Further research is needed to explore the trade-off between changes to EMS stroke care and call to hospital times and subsequent impacts on in-hospital care and patient outcomes.

**Supplementary Information:**

The online version contains supplementary material available at 10.1186/s12873-022-00713-6.

## Background

Stroke is responsible for a high global burden of mortality and disability [1]. In the UK there are over 80,000 new cases of stroke each year, it is the fourth leading cause of death and the single largest cause of acquired adult disability [2].

Around two thirds of acute stroke patients in England are brought to hospital by emergency medical services (EMS) [3]. Stroke care delivered by EMS clinicians has two primary foci: recognition, and rapid transport to specialist stroke care. Rapid processes are important because the availability and effectiveness of reperfusion treatments for ischemic stroke are time dependent [4]. Literature from around the globe has described EMS stroke care based on time intervals and reported on the importance of minimising times [5–8].

Despite awareness of the time critical nature of assessment, a recent UK national stroke audit (Sentinel Stroke National Audit Programme (SSNAP)) report identified that the time between onset of symptoms and arrival at hospital had increased by 50 minutes between 2013/14 and 2019/20 [9]. This time window includes the time between symptom onset and calling for help, determined by the patient and the people around them, and the time between calling EMS for help and arriving at hospital (call to hospital) which is determined by the healthcare system. Whilst English ambulance services publish mean call to hospital time for confirmed stroke patients as a quality indicator there is no set target this is judged against [10]. Haworth and McClelland [11] reported that median EMS call to hospital times, comprised of call to dispatch; dispatch to scene; on-scene time (OST); and leave scene to hospital, for stroke patients in one UK service increased by 27 minutes between 2011 and 2018.

Reversing this trend and reducing the call to hospital time for stroke patients should lead to improved patient outcomes. Therefore, this review aimed to identify and describe interventions devised to improve the efficiency of acute stroke care which reported an impact on ground-based EMS call to hospital times.

## Methods

A systematic review with narrative synthesis was conducted. The protocol for this review was registered with PROSPERO (reference CRD42021225603). The review is reported using the PRISMA and Synthesis Without Meta-analysis (SWiM) guidelines [12, 13].

### Search strategy and paper selection

The PICO model was used to structure the research question and a search strategy (see Additional file 1) was constructed to identify relevant papers:

- Population: suspected stroke patients attended by ground-based EMS.
- Intervention: interventions to reduce call to hospital times for suspected stroke patients.
- Comparator: standard, normal or previous call to hospital times for suspected stroke patients.
- Outcome: impact on call to hospital times for suspected stroke patients as a primary outcome. Impact on in-hospital times and processes is reported as a secondary outcome.

The search strategy was applied to the following databases either directly or using OVID or EBSCO through Newcastle University Library systems: Medline, EMBASE, CINAHL, the Cochrane Library and the Database of Research in Stroke (DORIS) from January 2000 to December 2020. The grey literature was searched using the first 200 hits on Google in April 2021. Reference list searches and citation chaining using Web of Science were done on included papers.

The title and abstract of all papers identified by the search strategy were independently screened by two reviewers (GM and SH). Papers were selected based on meeting all the following inclusion criteria:

- Any study design
- EMS or prehospital intervention
- Focus on stroke or suspected stroke patients
- Aim or objective includes reduction or improvement in EMS call to hospital or components of call to hospital time

Exclusion criteria:

- Papers prior to 2000 were excluded due to the changes in acute stroke care systems since this time
- Studies of Mobile Stroke Units (MSUs) and Helicopter EMS (HEMS) were excluded due to their specialist nature
- Papers focussed on public health interventions reducing time to call EMS
- Papers in languages other than English
- Letters, case studies and conference abstracts.

The full text of papers selected at the screening stage were independently reviewed by two reviewers (GM and SH). Conflicts at either stage were discussed with the wider team (TF and CP) if a consensus couldn’t be reached. EndNote (EndNote X9.2) was used to manage the literature search.

### Data extraction and risk of bias assessment

Data were extracted from included papers using a standardised form, developed for the study, by one reviewer (GM) with a second reviewer (SH) verifying data extraction on a random selection of 25% of included papers. The data extracted were: authors; year; country; sample size; study design; intervention; prehospital impact; and in-hospital impact. Included studies were assessed for risk of bias by one reviewer (GM) using either the Cochrane RoB2 [14] tool for randomised trials or the ROBINS-I [15] tool for non-randomised trials as appropriate. RoB2 produces a scale including low risk, some concerns or high risk. ROBINS-I produces a scale including low, moderate, serious, critical or no information.

### Data synthesis

Narrative synthesis was performed based on the SWiM guidelines as meta-analysis was felt to be inappropriate due to heterogeneity within the systems reported on and the range of measures used [13].

## Results

**Fig. 1:**
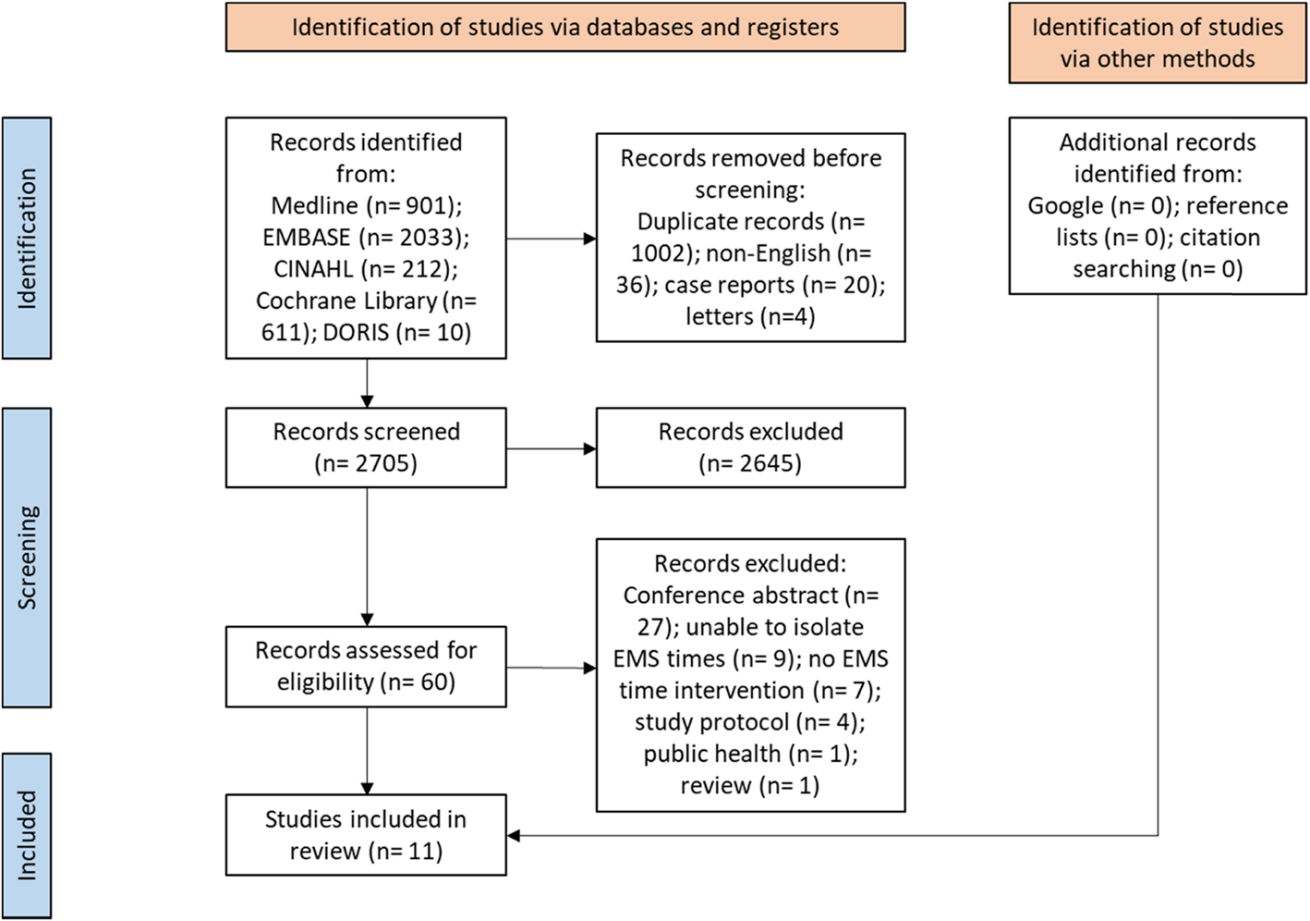
Flowchart summarising study screening and selection

The screening and selection processes are summarised in Fig. 1. From 3767 initial records 11 studies (16 − 26) were included in the review which are summarised in Table 1. Included studies were primarily before-and-after studies (*n* = 8, 73%) with suspected stroke patients attended by EMS as the primary population studied and most of the interventions described relied upon clinician education (*n* = 10, 91%). The studies included reported variable impacts on EMS call to hospital times with one study (9%) reporting a significant decrease in call to hospital time in one subgroup [16], two studies (18%) reporting a significant increase in call to hospital time [17, 18] and all other (73%) studies reporting no significant change [19–26].

**Table 1:** Summary of included studies

Lead author	Year	Country	Sample Size	Study design	Intervention	Prehospital impact	Risk of bias
*Studies targeting EMS dispatch +/− EMS clinicians*							
Berglund [16]	2012	Sweden	942 suspected stroke patients	RCT	Increased priority stroke dispatch and rapid transport to stroke unit. Meetings and education prior to study.	Dispatch randomised = shorter call to hospital but no change in OST. EMS randomised = no change.	Some concerns^a^
Mohamad [22]	2016	Denmark	476 stroke patients who received thrombolysis and/or thrombectomy	Before and after	EMS dispatch and paramedic training on large vessel occlusion scale and prioritisation	Reduced prehospital delay for thrombectomy but not for thrombolysis (non-significant)	Serious
Puolakka [25]	2016	Finland	77 thrombolysis candidates	Prospective cohort study	Dispatch of fire and rescue service to support ambulances with stroke patients	Non-significant decrease in OST	Serious
Watkins [26]	2013	UK	464 suspected or confirmed stroke patients	Interrupted time series	2-hour online training package for dispatchers	No change in call to hospital arrival pre and post implementation, improved recognition of stroke	Moderate
*Studies targeting EMS clinicians only*							
Frendl [20]	2009	USA	154 suspected stroke patients	Before and after	1-hour educational presentation with written material	No significant change in OST	Critical
Gorchs-Molist [17]	2020	Spain	17,135 suspected stroke patients	Before and after	6-hours online training	Increased call to hospital time with large % due to OST	Critical
Oostema [23]	2019	USA	1805 EMS transported patients	Interrupted time series	30-minute online training plus case-based feedback	Increased cases < 15 mins OST, no significant change in overall OST or transport time	Serious
Puolakka [24]	2016	Finland	289 thrombolysis candidates	Before and after	45-minute training session with interactive follow up group sessions	10% reduction in OST, no change in dispatch to hospital time	Moderate
*Studies targeting whole system change*							
De Luca [19]	2009	Italy	4895 suspected stroke patients	Cluster RCT	Training on stroke emergency care pathway	Reduced dispatch to hospital	Low^a^
Kendall [21]	2015	UK	351 thrombolysed patients	Before and after	Continuous quality improvement approach	No significant difference in call to door time	Critical
Wojner-Alexandrov [18]	2005	USA	1518 suspected stroke patients	Before and after	Monthly multilevel education sessions	Increased OST, transport and overall time	Critical

*EMS* Emergency medical services, *OST* On-scene time, *RCT* Randomised controlled trial.

^a^assessed using ROB2, all other studies assessed using ROBINS-I

### Risk of bias (ROB)

No studies were excluded based on ROB assessments (see Additional file 2), however the studies with the greatest ROB [17, 18, 20, 21] were all before and after studies reporting either no change or deterioration in call to hospital times whereas those with the lowest ROB [19, 24, 26] used more robust methods and reported either no change or an improvement in call to hospital times.

### Included studies

Included studies are described in three groups: studies targeting EMS dispatch +/− EMS clinicians; studies targeting EMS clinicians only; and studies targeting whole system change.

### Studies targeting EMS dispatch +/− EMS clinicians

Berglund et al. [16] described an RCT where the intervention included nurses working in the contact centre of a Swedish ambulance service, or paramedics physically assessing patients, randomising suspected stroke patients to either priority 2 (normal practice) or priority 1 (increased priority) to investigate impacts on the EMS system including time to arrival at a stroke unit. The intervention included ‘*meetings and education’* prior to the study. The results were based on 942 patients and showed increased priority by the contact centre reducing the call to hospital time by 13 minutes (*p* < 0.001) which comprised significant savings in call to dispatch, dispatch to arrival, departure to hospital, hospital to stroke unit and call to stroke unit but no reduction in OST. No significant changes were seen when paramedics randomised the patients. The intervention was also associated with more patients arriving at the stroke unit within 3 hours and increased rate of thrombolysis. This RCT was stopped early due to the lack of negative impact on the EMS system and the intervention was implemented into practice.

Mohamad et al. [22] described a simple intervention based on a four-question triage tool designed to aid dispatch and EMS staff to identify and prioritise large vessel occlusion (LVO) stroke patients for rapid transport to a centre with thrombectomy capabilities. This intervention showed no significant change in median call to hospital times for patients treated with thrombolysis, thrombectomy or both (pre-intervention 55 minutes vs post-intervention 56 minutes), however the in-hospital times for patients treated with thrombectomy significantly improved with no negative impact on prehospital or in-hospital times for patients treated with thrombolysis.

Puolakka et al. [25] described a novel intervention where dispatch of a fire service resource to support an ambulance attending a suspected stroke patient was hypothesised to reduce the OST. The study participants were thrombolysis candidates who were notified to the hospital neurologist. In this small study, no significant differences were found in any of the prehospital time metrics. The cases with fire support trended towards shorter times however this appears to be primarily influenced by the increased rate of high priority stroke dispatches in this population.

Watkins et al. [26] targeted a two-hour online educational package at EMS dispatchers based on preceding work in this area. This intervention was intended to improve dispatcher identification of stroke and look at the impact of identification on call to hospital times. The intervention resulted in a small, but non-significant, reduction in mean call to scene times (pre-intervention 12.2 minutes vs post-intervention 9.4 minutes, *p* = 0.068) and no change in mean call to hospital times (pre-intervention 45.0 minutes vs post-intervention 44.9 minutes, *p* = 0.23).

In summary, these studies which included EMS dispatch and clinicians used a mix of interventions but the only intervention that showed an important impact on call to hospital was increasing the priority from dispatch and throughout the whole system [16].

### Studies targeting EMS clinicians only

This second group of studies included four studies. Three studies with improved stroke recognition as an element of the intervention [17, 20, 23] and the only study purely focussed on reducing OST [24].

Frendl et al. [20] studied the impact on recognition and OST of a one-hour interactive educational presentation focused on stroke identification and the Cincinnati Prehospital Stroke Scale (CPSS) as part of a monthly educational program. There was a non-significant reduction of 2 minutes in the mean OST in the year following training compared to the year before.

Gorchs-Molist et al. [17] studied the impact of a six-hour online training package supplemented by an interactive forum and additional study resources focussed on an LVO stroke scale. The results included data on 17,135 suspected stroke patients across 4 years and showed an increase in call to hospital time of 4.7 minutes (*p* = 0.015) largely driven by increased OST (2.8 minutes, *p* = 0.034) potentially due to the use of the novel LVO scale.

Oostema et al. [23] described the development and implementation of a training package and personalised feedback as an intervention to improve stroke recognition and EMS compliance with metrics of quality stroke care including minimising OST. The study reported no change in median OST (pre-intervention 18 minutes vs post-intervention 17 minutes, *p* = 0.135) or overall transport times (pre-intervention 33 minutes vs post-intervention 34 minutes, *p* = 0.314) however the proportion of cases with short OST (≤15 minutes) significantly increased (pre-intervention 38% vs post-intervention 44%, *p* = 0.018). This intervention showed little impact on in-hospital processes apart from an improvement in the number of patients receiving thrombolysis within 45 minutes.

Puolakka et al. [24] reported the impact of a focussed training program involving a 45-minute training session and additional group sessions intended to reduce the OST to under 20 minutes. Data on thrombolysis candidates identified by EMS demonstrated a 10% reduction in median OST (pre-intervention 25 minutes vs post-intervention 22.5 minutes, *p* < 0.001) however this did not change the overall call to hospital time.

### Studies targeting whole system change

This third group of studies described whether interventions impacted on EMS times as part of a broad acute care system designed to efficiently get stroke patients to specialist care.

De Luca et al. [19] reported a cluster RCT from Italy studying the introduction of an emergency clinical pathway for EMS and emergency department personnel incorporating stroke identification using the CPSS, consideration of thrombolysis criteria and direct referral to the stroke unit. The intervention (described in Ferri et al. [27]) was delivered by facilitators training small groups [6–8] of EMS staff on the new pathway. The trial included 4895 suspected stroke patients and reported a four-minute reduction in mean call to hospital time in the intervention arm. This improvement was largely driven by changes in practice in central Rome whereas suburban areas reported increased dispatch to hospital times due to extended transportation distances. Patients at hospital were admitted to the stroke unit faster in the intervention arm and higher rates of thrombolysis were also reported.

Kendall et al. [21] described a continuous quality improvement project aimed at reducing delays to stroke thrombolysis involving ambulance and hospital services. The details of the intervention delivery are limited in the paper. There was no significant change in mean call to hospital time (pre-intervention 56.8 minutes vs post-intervention 57.5 minutes, *p* = 0.78) however the authors reported that ‘*despite the lack of beneficial effect specifically in call to door times (ie, prehospital times), we believe that the prehospital interventions of the Stroke 90 Project, such as prealert and cannulation in transit, will have contributed to more efficient running of in-hospital processes’.*

In the earliest paper included in the review Wojner-Alexandrov et al. [18] describe a program of EMS education in parallel with hospital and community education to improve stroke care. The EMS part of this study involved monthly education and the introduction of a modified Los Angeles Prehospital Stroke Scale (LAPSS). The intervention resulted in increased OST (pre-intervention 16.7 minutes vs post-intervention 18.2 minutes, *p* = 0.001), increased scene to hospital times (pre-intervention 15.6 minutes vs post-intervention 17.9 minutes, *p* = 0.001) which results in an increased overall call to hospital time (pre-intervention 42.2 minutes vs post-intervention 45.8 minutes, *p* = < 0.001). The study reported increased paramedic identification of stroke and variable impact on the six receiving hospitals in the study.

For all studies, Table 2 summarises the impacts of the interventions on the time spent in each phase by EMS. Positive figures indicate increased time spent in that phase and negative figures indicate time savings.

**Table 2:** Impact of interventions on phases of EMS care measured in minutes (+ increased time, − saved time)

Lead author	Comparator/baseline call to hospital	Call to scene	On Scene	Scene to hospital	Call to hospital
Studies targeting EMS dispatch +/1 EMS clinicians					
Berglund (EMD randomisation)	55	-6^a^	+ 1	-2^a^	−13^a^
Berglund (EMS randomisation)	45	-2	+ 1	0	+ 3
Mohamad	55				+ 1
Puolakka (FRS)	41	-1	−3	−1	−3
Watkins	45	−3			0
*Studies targeting EMS clinicians only*					
Frendl	NR		−2		
Gorchs-Molist	49	+ 1	+3^a^	+ 1	+5^a^
Oostema	NR		−1		
Puolakka	45	−1	-3^a^	0	−1
*Studies targeting whole system change*					
De Luca	36				−4
Kendall	57				+ 1
Wojner-Alexandrov	42	0	+2^a^	+2^a^	+4^a^

^a^indicates statistically significant result at *p* < 0.05. EMD = emergency medical dispatch, EMS = emergency medical services, FRS = fire and rescue services, NR = not reported

## Discussion

### General interpretation in context of other evidence

Based on the studies included in this review, education of EMS staff was the most common (*n* = 9/11) intervention component. The main patient group studied was the undifferentiated suspected stroke patients and most interventions showed little or no impact upon EMS times. Only four studies reported improvements in call to hospital times [16, 19, 24, 25], although only Berglund et al. [16] reported a significant reduction.

Included studies are described in three groups based on whether the study focussed on EMS dispatch, EMS clinicians or the whole acute stroke system. Dispatch focussed studies reported the most consistent impact on call to scene times whereas EMS clinician studies reported the most consistent impact on OST. Whole system studies reported mixed EMS impact, but all showed benefit across the whole acute pathway.

Call to scene is most likely to be affected by changes in dispatch behaviour and four studies focused on EMS dispatch [16, 22, 25, 26] which is an area that has received less attention than the delivery of face-to-face care by EMS clinicians. Reducing call to scene relies on dispatch identification of the patient as a stroke, or condition with equal high priority, and the response dictated by that priority of call. UK EMS are dispatched to acute stroke as a category 2 condition meaning it requires a blue-light response with a mean response time of < 18 minutes. Beyond upgrading the priority of dispatch to suspected stroke patients’, which may be difficult to justify given the known difficulties in identifying stroke during telephone triage [28], opportunities to improve the timeliness of responses may be limited due to the influence of factors like system pressures, distance and weather.

EMS OST might instinctively be the most modifiable of the time phases. However, the study by Puolakka et al. [24] was the only study focussed on reducing EMS OST times which reported a statistically significant improvement in OST although this didn’t impact the overall call to hospital time. The three other EMS clinician focussed studies included changes in stroke recognition in the intervention and there may be a trade-off between improving aspects of care such as recognition and the time taken. Simonsen et al. [29] reported that OST accounted for 44% of the total EMS time and the impact on OST shown by interventions in this review were variable and small (+/− 3 minutes).

There was variability in the phases of EMS time reported and in the EMS call to hospital performance in the comparator groups with times ranging from 36 to 57 minutes. This wide range of call to hospital times probably reflects variation in local practices and geographies and means that it is difficult to generalise the value of any intervention.

Interventions implemented in EMS to improve the quality of assessment may not show improvement in EMS metrics, and may even negatively impact on EMS times, but may positively impact on in-hospital metrics demonstrating the need to examine the whole acute pathway. If longer EMS times are linked to direct access to specialist care at regional centres which in turn leads to better patient outcomes then this is a worthwhile trade-off [30]. Previously, simple actions by EMS like pre-notification were shown to positively impact on quality metrics in receiving hospitals [4]. A meta-analysis by Huang et al. [31] reported that EMS education and training programs could increase the rate of thrombolysis in hospital and that EMS pre-notification increased the rate and speed of thrombolysis. Six studies in this review [16, 18, 19, 21–23] included data on various in-hospital metrics and all reported improvements in hospital-based care.

### Limitations of the evidence included

Most papers included were assessed to be at high levels of bias with regards to the outcome of interest, largely due to a lack of detail about intervention components, delivery or how uptake and impact was measured. These missing data make replicating the studies, assessing the quality or building on the results difficult. In addition, few included studies’ primary focus was on reducing EMS times, most studies included other objectives or interventions such as introducing new assessment tools or redirection policies. Whilst there was no formal measure of heterogeneity, it was clear that the included studies were from diverse settings, targeting heterogeneous patient populations and reporting different outcome measures.

Four studies [21, 22, 24, 25] defined their sample population by receipt of, or eligibility for, reperfusion therapy (thrombolysis and thrombectomy) whereas the other seven studies included the wider suspected stroke population. Using a retrospectively identified population defined by a hospital-based intervention hinders generalisation of the impact to the wider EMS suspected stroke population.

### Limitations of the review process

Selection of studies for inclusion was challenging with many studies either reporting prehospital times but not specifically targeting them with an intervention or reporting onset to hospital times such that isolating the EMS times was not possible. The restriction to English language papers means some relevant papers published in other languages may have been missed.

Trial registries were not searched so ongoing studies may have been overlooked. Data extraction and risk of bias assessment were largely done by a single author which may have introduced a personal bias.

Conference abstracts with information relevant to the review were identified during the screening process and related full text papers were searched for. Exclusion of abstracts without full papers may have excluded some further evidence.

This review focussed on interventions targeting traditional ground-based ambulance services, and so excluded studies of HEMS and MSUs. Both HEMS and MSUs may be able to reduce the time between patients calling for help and accessing definitive care but these are limited resources and evidence of their impact is not scalable in the same fashion as the interventions included in the review.

### Implications for practice, policy and future research

Interventions were often described as single instances as evidenced by the high number of before and after studies. Future evaluation may need to consider more sustained and multi-site efforts supported by implementation science approaches such as normalisation process theory [32], that focus on how collaborative work is enacted effectively in practice, to deliver meaningful change. Studies trying to identify the impact of any interventions in this area should recognise and control for any underlying trends in call to hospital times or the individual phases targeted. The 5 minute change in time reported in one study [17] needs to be considered in the context of wider systematic changes over the 4 year time frame. Further research may be needed to better understand why baseline times are increasing and what factors are contributing to this in order to appropriately target interventions. Practice, policy and future research in this area needs to consider the whole acute stroke patient journey from onset of symptoms, initial call for help, acute treatment in hospital through to longer term patient related outcomes as EMS focussed interventions may not immediately show an impact. Ongoing research of novel interventions in EMS stroke care such as portable diagnostics, and remote assessment technologies such as telemedicine [33], should report the impact on prehospital times in the context of their role in the patient pathway e.g. identification of patients potentially suitable for thrombectomy.

## Conclusions

The studies identified described interventions intended to enhance the acute stroke pathway, but EMS stroke times were not usually improved. Future research needs to consider the impact of changes to EMS stroke care, not just on call to hospital times but on patient outcomes judged by metrics across the whole patient journey.

## Supplementary Information

**Additional file 1.** (Prehospital stroke time lit review add file 1.doc) includes the search terms.

**Additional file 2.** (Prehospital stroke time ROB table) includes the risk of bias assessments.

## Data Availability

All data generated or analysed during this study are included in this published article and its supplementary information files.

## Abbreviations

CPSS: Cincinnati Prehospital Stroke Scale
EMD: Emergency Medical Dispatch
EMS: Emergency Medical Services
HEMS: Helicopter Emergency Medical Services
LAPSS: Los Angeles Prehospital Stroke Scale
LVO: Large Vessel Occlusion
MSU: Mobile Stroke Unit
OST: On-scene Time
RCT: Randomised Controlled Trial
ROB: Risk of Bias
SSNAP: Sentinel Stroke National Audit Project
UK: United Kingdom
