# Supplementary material for: How do interventions to improve the efficiency of acute stroke care affect prehospital times? A systematic review and narrative synthesis
Source: BMC Emerg Med. 2022 Sep 3;22:153. doi: 10.1186/s12873-022-00713-6 (PMC9440533; doi:10.1186/s12873-022-00713-6)
Supplement: Supplementary file 1 — Additional file 1. (Prehospital stroke time lit review add file 1.doc) includes the search terms. [file 12873_2022_713_MOESM1_ESM.docx]

**How do interventions to improve the efficiency of acute stroke care affect prehospital times? A systematic review and narrative synthesis – additional file 1: search terms**

**Authors**

Graham McClelland, Sarah Hepburn, Tracy Finch, Christopher I. Price

**Medline ® 1946 to December Week 2 2020:**

1. Exp Stroke/di or cerebrovascular disorders/di or exp brain ischemia/di or stroke.mp or cerebrovascular accident.mp or CVA.mp
2. Exp emergency medical services/ or exp emergency medical technician/ or paramedic*.mp or ambulance*.mp or prehospital.mp or pre-hospital.mp or EMS.mp or emergency medical*.mp or out of hospital.mp
3. Time*.mp or timing*.mp or delay*.mp
4. Intervention*.mp or trial*.mp or project*.mp or program*.mp
5. 1 and 2 and 3 and 4 (Filtered by 2000+)

**EMBASE 1996 to 2020 Week 50**

1. Exp Stroke/di or cerebrovascular disorders/di or exp brain ischemia/di or stroke.mp or cerebrovascular accident.mp or CVA.mp
2. Exp emergency medical services/ or paramedic*.mp or ambulance*.mp or prehospital.mp or pre-hospital.mp or EMS.mp or emergency medical*.mp or out of hospital.mp
3. Time*.mp or timing*.mp or delay*.mp
4. Intervention*.mp or trial*.mp or project*.mp or program*.mp
5. 1 and 2 and 3 and 4 (Filtered by 2000+)

**CINAHL**

(MH "Stroke+/DI" OR "CVA" OR "CEREBROVASCULAR ACCIDENT") AND (MH "Emergency Medical Services+" OR "PARAMEDIC" OR "AMBULANCE" OR "EMS" OR "PREHOSPITAL" OR "PRE-HOSPITAL") AND (MH "Time+" OR "DELAY" OR "TIMING") AND (intervention or trial or project or program) AND 2000+ AND abstracts available

**Cochrane library**

(Stroke OR CVA OR cerebrovascular accident) AND (paramedic OR ambulance OR EMS OR Emergency medical services) AND (time OR timing OR delay) AND (intervention or trial or project or program)

Limited to 2000+

**Ask DORIS**

Combinations of ambulance, paramedic, time, delay
